# Hyper-Mol: Molecular Representation Learning via Fingerprint-Based Hypergraph

**DOI:** 10.1155/2023/3756102

**Published:** 2023-02-01

**Authors:** Shicheng Cui, Qianmu Li, Deqiang Li, Zhichao Lian, Jun Hou

**Affiliations:** ^1^School of Cyber Science and Engineering, Nanjing University of Science and Technology, Nanjing, China; ^2^School of Computer Engineering, Nanjing Institute of Technology, Nanjing, China; ^3^School of Intelligent Manufacturing, Wuyi University, Jiangmen, China; ^4^School of Computer Science, Nanjing University of Posts and Telecommunications, Nanjing, China; ^5^School of Social Science, Nanjing Vocational University of Industry Technology, Nanjing, China

## Abstract

With the development of artificial intelligence (AI) in the field of drug design and discovery, learning informative representations of molecules is becoming crucial for those AI-driven tasks. In recent years, the graph neural networks (GNNs) have emerged as a preferred choice of deep learning architecture and have been successfully applied to molecular representation learning (MRL). Up-to-date MRL methods directly apply the message passing mechanism on the atom-level attributes (i.e., atoms and bonds) of molecules. However, they neglect latent yet significant hyperstructured knowledge, such as the information of pharmacophore or functional class. Hence, in this paper, we propose Hyper-Mol, a new MRL framework that applies GNNs to encode hypergraph structures of molecules via fingerprint-based features. Hyper-Mol explores the hyperstructured knowledge and the latent relationships of the fingerprint substructures from a hypergraph perspective. The molecular hypergraph generation algorithm is designed to depict the hyperstructured information with the physical and chemical characteristics of molecules. Thus, the fingerprint-level message passing process can encode both the intra-structured and inter-structured information of fingerprint substructures according to the molecular hypergraphs. We evaluate Hyper-Mol on molecular property prediction tasks, and the experimental results on real-world benchmarks show that Hyper-Mol can learn comprehensive hyperstructured knowledge of molecules and is superior to the state-of-the-art baselines.

## 1. Introduction

Machine learning has achieved great success in the field of artificial intelligence (AI), which has been pervasively adopted in many human-centered applications [1–5]. Following the machine learning rules, large amounts of research effort have been dedicated to develop new paradigms for drug design and discovery in recent years. How to learn informative representations of molecules is critically important for AI-driven tasks [6–8]. For example, the well-learned molecular representations can be beneficial for molecular property prediction, which advances drug candidate selection for further validation and virtual screening on large datasets.

The chemical fingerprints [9] are widely used for representing molecules, the algorithms of which normally encode the physical or chemical characteristics of molecules into bit vectors. Another pipeline of research [10–12] introduces deep learning [13] to generate structure-aware or context-aware neural fingerprints for molecules. Since molecules can be naturally converted to graphs, where atoms and bonds are represented as nodes and edges, respectively [14], graph neural networks (GNNs) are commonly applied for molecular representation learning (MRL). Most related approaches [15–19] have dedicated tremendous effort on modeling atom-level relationships. Some [6–8, 14, 20] utilize the molecular geometry and structural information to develop a self-supervised learning paradigm for pretraining the GNN models. Following the message passing rules of GNNs, they carefully design the learning procedures to encode structural information on atom and bond attributes. Despite the promising results achieved by recent MRL methods in many drug design and discovery tasks, we argue that the following issues have not been solved.

- The chemical fingerprints use bits to preserve the existence of some physical or chemical characteristics of molecules. However, the topological information and the latent relationships among the extracted fingerprint substructures cannot be leveraged in such bit-style forms.
- Although some structure-aware or context-aware information about atom and bond interactions can be encoded to generate molecular representations, the hyperstructured knowledge, such as the information of a pharmacophore or functional class, has not been exploited.

Hence, to deal with the aforementioned problems, we introduce the concept of hypergraph and propose a novel MRL framework, dubbed Hyper-Mol, which encodes fingerprint-based **Hyper**graph structures of **Mol**ecules via GNNs. Hyper-Mol further exploits the information underneath the bit-style molecular fingerprints, learning molecular representations by exploring the hyperstructured knowledge and the latent relationships of the fingerprint substructures. Specifically, in Hyper-Mol, we utilize molecular fingerprint algorithms to produce topological fingerprints with physical and chemical characteristics of molecules, in which the pharmacophore-aware or functional class-aware components can be embedded in the generated clusters (i.e., the substructures of fingerprints) according to the algorithms. The basic idea of molecular hypergraph generation is that two objects are close to each other if they are referenced by similar or shared objects [2, 21, 22]. Thus, the hypergraph of each molecule is then constructed based on the topological relationships among the fingerprint substructures. To be precise, *any two fingerprint substructures of a molecule that have overlapped subregions (i.e., shared atoms or bonds) should be close to each other in the hypergraph*, which means that they will have a positive hyperlink in the hypergraph. The intra-structured information in fingerprint subgraphs and the inter-structured information in fingerprint hypergraphs are encoded via the message passing mechanism to learn comprehensive hypergraph representations for molecules.

Hence, we conclude our contributions as follows:

1. We propose Hyper-Mol, which learns molecular representations by utilizing molecular fingerprints from a hypergraph perspective.
2. The algorithm of molecular hypergraph generation is designed for preserving the hyperstructured information with physical and chemical characteristics of molecules.
3. The hyperstructured knowledge of molecular fingerprints can be exploited by the fingerprint-level message passing process from both intra-structured and inter-structured information according to the molecular hypergraphs.
4. The experimental results show that Hyper-Mol can learn comprehensive molecular representations for molecular property prediction tasks compared with the state-of-the-art methods.

The rest of the paper is organized as follows: In Section 2, related work is briefly introduced. In Section 3, we present Hyper-Mol. After that, the proposed method is evaluated over several state-of-the-art baselines and the detailed experiments are given in Section 4. Finally, we conclude our work and point out the future work in Section 5.

## 2. Related Work

### 2.1. Fingerprint Generation on Molecules

Traditional ways of representing molecules are the chemical fingerprints, such as pharmacophore fingerprints [23, 24], functional-class fingerprints, and extended-connectivity fingerprints [9]. These algorithms mostly utilize bit vectors to represent the existence of pharmacophore, functional classes, or geometric characteristics in molecules. Inspired by the success of deep learning in computer vision and natural language processing, some deep neural architectures are introduced to generate low-dimensional vector representations for molecules. For example, prior studies [10, 11] make use of the convolutional neural networks [25] to learn molecular neural fingerprints. Xu et al. [12] propose Seq2Seq fingerprints by exploiting the SMILES [26, 27] strings based on the sequence-to-sequence neural framework [28, 29].

### 2.2. Molecular Representation Learning on Graphs

Due to the fact that molecules can be easily converted to graph data, graph neural networks (GNNs) have been widely adopted to learn molecular representations in recent years. Some approaches [15–17] apply graph convolutional networks [30] to encode atom relationships in molecules. To capture bond features, [18, 19] further develop the message passing process that also models bond interactions. MGCN [31] is proposed to model the multilevel quantum interactions of molecules from hierarchical perspectives (i.e., atom-wise, pair-wise, triple-wise, and so on). With the development of self-supervised learning, Hu et al. [20] propose pretraining strategies to learn molecular representations with self-supervised pretext tasks in atom level. They define several types of graph proximity as the self-supervised learning objectives, which push GNNs to generate meaningful atom representations. Other up-to-date techniques [6–8, 14] follow the same idea and develop more molecular information-related pretext strategies. N-Gram [32] conducts node (atom) representations by predicting the node (atom) attributes, which utilizes SMILES strings.

Different from the previous work, our proposed Hyper-Mol not only enhances the expressive power of chemical fingerprints but also models the topological information and the relationships of the fingerprint substructures (with physical and chemical characteristics) from a hypergraph perspective.

## 3. Hyper-Mol

### 3.1. Preliminaries

Let *G*=(*V*, *E*) be a molecular graph, where *V* denotes the atom set and *E* denotes the bond set of the molecule. Suppose a molecule has *n* fingerprint substructures and the structural set is *𝒮*={*S*_1_, *S*_2_,…, *S*_*n*_}. *S*_*i*_=(*V*_*i*_, *E*_*i*_), where 1 ≤ *i* ≤ *n* and *V*_*i*_ ⊂ *V*, *E*_*i*_ ⊂ *E*.

#### 3.1.1. Molecular Fingerprint

Molecular fingerprints are a way of encoding the structure of a molecule [33]. The most common type of fingerprint is a series of binary digits (bits) [34, 35] that represent the presence or absence of particular substructures in the molecule. Therefore, the similarity between two molecules can be calculated by comparing their fingerprints.

#### 3.1.2. Graph Neural Networks

The architecture of graph neural networks (GNNs) has recently been developed as one of the crucial deep learning techniques. The core idea behind GNNs is message passing through network topology in graphs. Node representations are updated by propagating and aggregating structural information from the neighborhood to the target node.

(1) $$\begin{array}{c} a_{v}^{\left(k\right)}=AGGREGATE\left(\left\{h_{u}^{\left(k-1\right)}:u\in N\left(v\right)\right\}\right)\text{,}\\ h_{v}^{\left(k\right)}=COMBINE\left(h_{v}^{\left(k-1\right)},a_{v}^{\left(k\right)}\right)\text{,}\\ h_{G}^{\left(k\right)}=READOUT\left(\left\{h_{v}^{\left(k\right)}:v\in V\right\}\right)\text{,} \end{array}$$

where the AGGREGATE function in the *k*^th^ layer aggregates neighborhood information of the target node *v*, and the COMBINE function combines the information of the target node *v* and its neighborhood *𝒩*(*v*). The READOUT function normally applies sum/mean/max pooling methods to generate the graph representation *h*_*G*_.

### 3.2. Overall Framework

**Figure 1:**
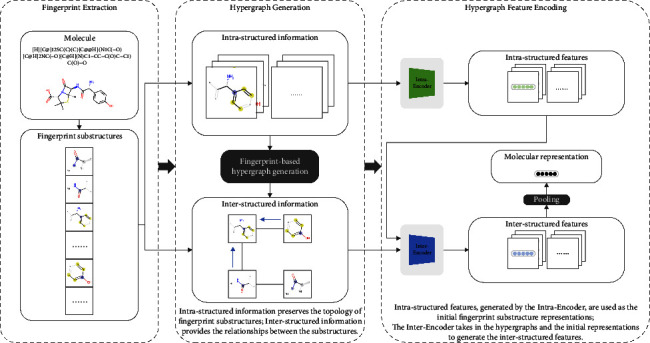
An illustration of the Hyper-Mol framework.

Hyper-Mol encodes graph structures of molecules via the fingerprint-based features. As illustrated in Figure 1, the overall framework of Hyper-Mol consists of three main components: fingerprint extraction, hypergraph generation, and hypergraph feature encoding.

#### 3.2.1. Fingerprint Extraction

The extended-connectivity fingerprints (ECFPs) are a class of topological fingerprints for molecular characterization [9]. Physical and chemical characteristics of molecules can be encoded by ECFPs. For example, the functional-class fingerprints are a variant of the ECFPs that describe substructures according to their roles in pharmacophores. Thus, in Hyper-Mol, we employ the ECFPs algorithm to extract molecular fingerprints (note that any fingerprint extraction algorithms that satisfy the rules of Hyper-Mol can be employed without restriction) due to its interpretability and effectiveness in modeling [36].

#### 3.2.2. Hypergraph Generation

The hypergraph of each molecule is then generated based on the topological relationships among the extracted fingerprint substructures and the molecular graph, where nodes are the fingerprint substructures and edges are the connections between substructures in the molecular graph. To be precise, the intra-structured information of a fingerprint substructure is composed of atoms and bonds. Any two substructures that have overlapped intra-structured regions (i.e., shared atom-level structures) in the molecular graph will have a hyperlink between each other.

#### 3.2.3. Hypergraph Feature Encoding

In Hyper-Mol, the Intra-Encoder encodes the intra-structured information for each fingerprint substructure, the output of which is used as the initial fingerprint substructure representations. The Inter-Encoder takes in the hypergraphs and the fingerprint substructure representations of molecules afterwards, propagating and aggregating the inter-structured information among fingerprint substructures following the message passing mechanism of GNNs. Based on equation (1), the hypergraph-level representations of molecules are obtained after training the neural models.

### 3.3. Fingerprint-Based Hypergraph Generation

The extended-connectivity fingerprints are circular topological fingerprints that are designed for molecular characterization and structure-activity modeling. In the hypergraph generation process, we first apply the ECFPs algorithm [9] to generate fingerprints and the substructures.

(2) $$\begin{array}{c} \left\{S_{1},\ldots ,S_{M}\right\}=ECFP\left(\left\{G_{1},\ldots ,G_{M}\right\}\right)\text{.} \end{array}$$

Suppose there are *M* fingerprints generated according to *M* molecules. *𝒮*={*S*_1_, *S*_2_,…, *S*_*n*_} denotes the substructure set of a fingerprint from molecular graph *G* (without loss of generality, we omit the subscripts of *S*, *G* for simplicity). Algorithm 1 illustrates the process that generates the hypergraph of a molecule based on its fingerprint substructures. We first obtain all the relative positions among the fingerprint substructures by the Cartesian product (Line 2). And then, we set a positive hyperlink between the two substructures if they share at least one common subregion from *G* (Line 5 to 6). Otherwise, a negative hyperlink will be set between the two (Line 8). *ℰ* collects all the hyperlinking information among the fingerprint substructures (Line 10). Finally, a new hypergraph of the molecule is generated.

### 3.4. Hypergraph Feature Encoding

Hyper-Mol encodes hypergraph features by the two kinds of encoders: the Intra-Encoder and the Inter-Encoder.

#### 3.4.1. Intra-Encoder

According to the ECFPs algorithm, the number of the generated fingerprint substructures is fixed. Thus, the Intra-Encoder simply adopts the one-hot encoding to distinguish each fingerprint substructure in a “fingerprint substructure vocabulary” from every other fingerprint substructure in the “vocabulary.” The output representations *X* of fingerprint substructures are a *N* × *N* matrix, where *N* represents the number of fingerprint substructures and also the fixed length of the one-hot vector. Each vector in the matrix consists of 0 s in all cells with the exception of a single 1 in a cell used uniquely to identify the fingerprint substructure.

#### 3.4.2. Inter-Encoder

The molecular hypergraphs and the one-hot fingerprint substructure representations are fed to the Inter-Encoder, in which we apply two widely-adopted GNN backbones, i.e., the graph convolutional networks (GCNs) and graph isomorphism networks (GINs), to respectively encode the hyperstructured features for each molecule.

The layer-wise propagation rule of GCNs in the Inter-Encoder is as follows:

(3) $$\begin{array}{c} H^{\left(k+1\right)}=\sigma \left({\tilde{D}}^{-\left(1/2\right)}\tilde{A}{\tilde{D}}^{-\left(1/2\right)}H^{\left(k\right)}W^{\left(k\right)}\right)\text{,}\\ H^{\left(0\right)}=X\text{,} \end{array}$$

where $\tilde{A}=A+I_{n}$ is the adjacency matrix of the undirected hypergraph *𝒢* with added self-loops. *I*_*n*_ is the identity matrix. ${\tilde{D}}_{ii}=\underset{j}{\sum }{\tilde{A}}_{ij}$. *W*^(*k*)^ is the *k*^*th*^ layer trainable weight matrix and *σ*(·) is an activation function. *H*^(*k*)^ represents the hidden representations of the fingerprint substructures in the *k*^*th*^ layer.

Different from GCN, GIN generalizes the Weisfeiler–Lehman test and achieves maximum discriminative power among GNNs. The multilayer perceptrons are employed to update the representations of fingerprint substructures in the GIN layer-wise propagation process.

#### 3.4.3. Hypergraph Representation

To obtain the hypergraph representation of *𝒢*, we apply a sum-pooling layer after the graph convolution layers of Inter-Encoder.

### 3.5. Time Complexity

Given a molecular graph *G*=(*V*, *E*) and its generated hypergraph *𝒢*=(*𝒮*, *ℰ*), the time complexity of extracting fingerprint substructures is *𝒪*(|*V*|^2^) following the ECFPs algorithm that two iterations are enough for fingerprints to be functional in similarity search and clustering [9]. With the complexity of *𝒪*(|*𝒮*|), we can obtain the nodes (i.e., the fingerprint substructures) of the molecular hypergraph. After that, the edge (i.e., the hyperlink) generation in the hypergraph can be operated in *𝒪*((1/2)|*𝒮*|^2^). Due to the GNN architecture, the time complexity of graph convolution operation is *𝒪*(|*ℰ*|) per neural layer.

## 4. Experiments

To evaluate the performance of Hyper-Mol, we compare it with multiple state-of-the-art baseline methods on various molecular property prediction tasks, such as bioactivity, pharmacokinetics and toxicity. The whole framework is implemented based on PyTorch (https://pytorch.org/), DGL (https://www.dgl.ai/), DGL-LifeSci (https://lifesci.dgl.ai/), and RDKit (https://www.rdkit.org/).

### 4.1. Datasets

We conduct the experiments on the HIV, BBBP, BACE, Tox21, SIDER, and ClinTox molecular property prediction benchmark datasets (https://moleculenet.org/datasets-1), all of which are from MoleculeNet [37]. The prediction tasks can be formulated as a set of binary and multilabel graph-level classification problems. To be precise, the HIV, BBBP, and BACE datasets are used for the binary classification tasks and the Tox21, SIDER, and ClinTox datasets are for the multilabel classification tasks. The detailed descriptions of all datasets are shown in Table 1.

### 4.2. Baselines

We thoroughly evaluate Hyper-Mol against 6 state-of-the-art approaches. Among them, graph convolutional networks (GCN) [30] and graph isomorphism networks (GIN) [38] are the two popular GNN-based frameworks that can learn the structural information of network-based data in a supervised manner. N-Gram [32] extracts the context of vertices and assembles the representations in short walks directly through the molecule graph. Hu et al. [20] design self-supervised strategies for learning molecular representations. SchNet [16] is a continuous-filter convolutional neural network for modeling quantum interactions and MGCN [31] considers modeling bond features in message passing processes.

### 4.3. Experimental Settings

As suggested in the previous work [20], we adopt the scaffold split to create the train/validation/test with the ratio of 8 : 1 : 1. The scaffold splitting method splits molecules according to molecular substructures, which is more challenging yet realistic. Compared with the random split, it can better evaluate the generalization ability of the models on out-of-distribution data samples.

We apply the GCN and GIN architectures (i.e., the AGGREGATE and COMBINE functions) in Hyper-Mol, respectively. The sum pooling is used as the READOUT function to obtain the molecular graph representations. We train the neural networks with 100 epochs and the batch size is 32 in each epoch. ReLU [39] is adopted as the activation function, and Adam [40] is employed for optimization. To fit the supervised molecular property prediction tasks, we use the sigmoid function and the binary cross entropy to measure the loss between the target and the predicted probabilities. Since the input vectors of the fingerprint representations are generated by the ECFPs algorithm, we set the two hyperparameters (i.e., the length and the radius) of ECFPs with commonly-adopted default values 2048 and 2, respectively.

We use the ROC-AUC (area under the receiver operating characteristic curve) [41] as the evaluation metric for both the binary and multilabel classification tasks. We execute three independent runs and the mean and the standard deviation of test ROC-AUC on each benchmark are reported.

### 4.4. Results

#### 4.4.1. Overall Performance

Tables 2 and 3 summarize the overall performances of Hyper-Mol along with other baseline methods, where the best results (i.e., higher is better) are shown in bold. We have the following observations: (1) Hyper-Mol achieves the best average ROC-AUC scores in both binary and multilabel tasks over the experimented datasets. Besides, Hyper-Mol outperforms all the state-of-the-art baselines on 4/6 datasets; (2) the GCN backbone in the Hyper-Mol framework is more effective than the GIN, which achieves an overall relative improvement of 1% on the average ROC-AUC scores.

#### 4.4.2. Contribution of Hyper-Mol in Binary Classification

As present in Tables 2 and 4, Hyper-Mol surpasses all the methods on the BBBP and BACE datasets, and also shows rival performance compared with the best-performed N-Gram on the HIV dataset. Moreover, both the GCN and GIN backbones in Hyper-Mol with fingerprint-level message passing mechanism achieve 21.1% and 23.3% improvement, respectively, in comparison with those in the atom level.

#### 4.4.3. Contribution of Hyper-Mol in Multilabel Classification

Tables 3 and 4 demonstrate that the multilabel classification tasks are more challenging than the binary ones. The models proposed by Hu et al. and N-Gram perform competitive in the multilabel classification tasks. Hyper-Mol still achieves the highest results on the SIDER (with 27 tasks) and ClinTox (with 2 tasks) datasets, respectively. As the similar phenomenon observed in binary classification tasks, the fingerprint-level message passing processes in Hyper-Mol applying the GCN and GIN backbone neural architectures also achieve 21.8% and 19.3% improvement, respectively, compared with the atom-level message passing.

#### 4.4.4. Impact of ECFPs Hyperparameters

**Figure 2:**
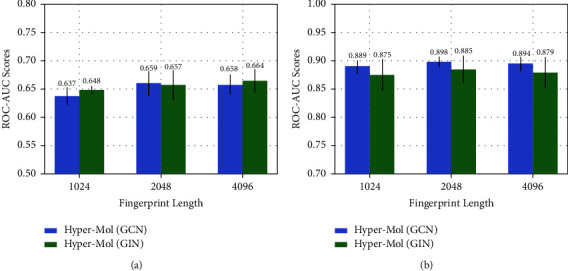
The impact of fingerprint length. (a) SIDER dataset (radius = 2). (b) BACE dataset (radius = 2).

**Figure 3:**
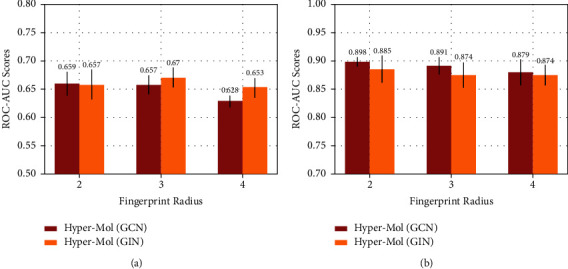
The impact of fingerprint radius. (a) SIDER dataset (length = 2048). (b) BACE dataset (length=2048).

Hyper-Mol applies the ECFPs algorithm to generate fingerprints for molecules. To show the impact of the hyperparameters (i.e., the length and the radius) on Hyper-Mol, we conduct two types of model sensitivity experiments: (1) we fix the radius with 2, and vary the length in the set {1024,2048,4096}; (2) we vary the radius from 2 to 4, with the length = 2048 fixed.  Figure 2 presents how the fingerprint length affects the performance of Hyper-Mol on the SIDER (multilabel task) and BACE (binary task) datasets, respectively, under the circumstance that the radius is set to 2. We can observe that with a larger fingerprint length, Hyper-Mol with both GCN and GIN backbones achieves better performance on the SIDER dataset. The best ROC-AUC score achieved by Hyper-Mol (GIN) with length = 4096 reaches to 0.664 ± 0.021. On the BACE dataset, there is also an improvement achieved by the larger length (2048 and 4096) compared with the relative small length (1024).  Figure 3 offers the observation that with the fixed length = 2048 of fingerprints, the larger radius (4) shows a negative effect on the performance of Hyper-Mol compared with the relative small radius (2 and 3) on both the SIDER and BACE datasets.

### 4.5. Discussion

Overall, encoding fingerprint-based features from a hypergraph perspective provides a powerful solution for learning molecular graph representations. Results on the experimented datasets show that the proposed Hyper-Mol is superior to the state-of-the-art baseline methods on the molecular property prediction tasks. The message passing processes in the baselines aggregate and propagate structural information in the atom level, which force their neural networks to learn relatively “microscopic” graph-structured knowledge of molecules, i.e., the relationships of atoms and bonds. However, the more sophisticated information of molecules, such as the pharmacophore-aware or functional class-aware characteristics, is normally embedded in some meaningful clusters of atoms and bonds, for example, the components of molecular fingerprint substructures. Different from the ways of atom-level message passing that lack meaningful “interactions” between clusters, Hyper-Mol perceives hyperstructured information through the fingerprint-level message passing mechanism. Instead of absorbing atom-attributed or bond-attributed features only, Hyper-Mol utilizes fingerprint-attributed features to depict informative context relationships of the molecular fingerprint substructures. Physical and chemical characteristics of fingerprint-specific knowledge can be encoded into the final molecular graph representation from a hypergraph perspective. Therefore, the overall performance of Hyper-Mol is superior to the baselines.

## 5. Conclusions and Future Work

In order to learn molecular representations with more sophisticated knowledge of physical and chemical characteristics, we propose Hyper-Mol, a novel MRL framework, which encodes **Hyper**graph structures of **Mol**ecules via fingerprint-level message passing mechanism. Hyper-Mol constructs hypergraphs of molecules by utilizing both intra-structured and inter-structured topological information of chemical fingerprint substructures, and applies GNNs to learn meaningful molecular representations based on the extracted hyperstructured features. Experimental results present that Hyper-Mol can depict informative context relationships of the fingerprint substructures and is superior to the state-of-the-art approaches on various molecular property prediction tasks, such as bioactivity, pharmacokinetics and toxicity.

Future work would focus on exploring self-supervised or unsupervised learning framework for encoding hypergraph knowledge of molecules. Meanwhile, we also consider to incorporate both atom-level and fingerprint-level information to learn more comprehensive representations for molecules.

## Figures and Tables

**Algorithm 1:**
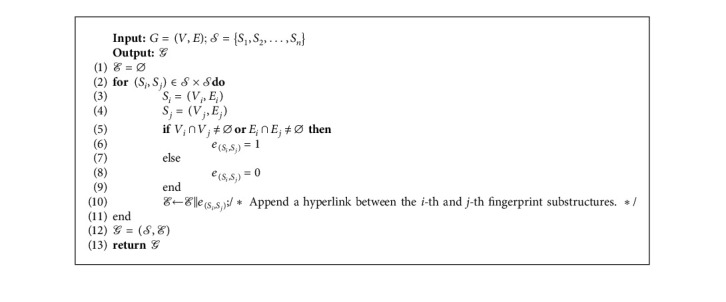
Algorithm 1: Hypergraph generation algorithm based on fingerprint substructures.

**Table 1:** Detailed descriptions of the experimented datasets.

Datasets	# Molecules	# Tasks	Description
HIV	41127	1	For the prediction of the ability of the tested compounds to inhibit HIV replication
BBBP	2039	1	For the modeling and prediction of the barrier permeability
BACE	1513	1	For the prediction of binding results for a set of inhibitors of human beta-secretase 1
Tox21	7831	12	For measuring the toxicity of the tested compounds
SIDER	1427	27	For the prediction of grouped drug side-effects
ClinTox	1478	2	For the prediction of clinical trial toxicity (or absence of toxicity) and FDA approval status

**Table 2:** The test ROC-AUC performance of different models in binary classification benchmarks.

Datasets	HIV	BBBP	BACE	Avg.
GCN	0.740 ± 0.030	0.718 ± 0.009	0.716 ± 0.020	0.725
GIN	0.753 ± 0.019	0.658 ± 0.045	0.701 ± 0.054	0.704
*N*-Gram	**0.830** ± **0.013**	0.912 ± 0.030	0.876 ± 0.035	0.873
Hu et al.	0.802 ± 0.009	0.708 ± 0.015	0.859 ± 0.008	0.790
SchNet	0.702 ± 0.034	0.848 ± 0.022	0.766 ± 0.011	0.772
MGCN	0.738 ± 0.016	0.850 ± 0.064	0.734 ± 0.030	0.774

Hyper-Mol (GCN)	0.814 ± 0.011	**0.922** ± **0.012**	**0.898** ± **0.009**	**0.878**
Hyper-Mol (GIN)	0.808 ± 0.016	0.910 ± 0.022	0.885 ± 0.024	0.868

The numbers in bold represent the best performance.

**Table 3:** The test ROC-AUC performance of different models in multilabel classification benchmarks.

Datasets	Tox21	SIDER	ClinTox	Avg.
GCN	0.709 ± 0.026	0.536 ± 0.032	0.625 ± 0.028	0.623
GIN	0.740 ± 0.008	0.573 ± 0.016	0.580 ± 0.044	0.631
*N*-Gram	0.769 ± 0.027	0.632 ± 0.005	0.855 ± 0.037	0.752
Hu et al.	**0.787** ± **0.004**	0.652 ± 0.009	0.789 ± 0.024	0.743
SchNet	0.772 ± 0.023	0.539 ± 0.037	0.715 ± 0.037	0.675
MGCN	0.707 ± 0.016	0.552 ± 0.018	0.634 ± 0.042	0.631

Hyper-Mol (GCN)	0.742 ± 0.038	**0.659** ± **0.021**	0.875 ± 0.078	**0.759**
Hyper-Mol (GIN)	0.723 ± 0.042	0.657 ± 0.026	**0.879** ± **0.056**	0.753

The numbers in bold represent the best performance.

**Table 4:** The test ROC-AUC performance of different GNN backbones with atom-level and fingerprint-level structural information.

Tasks	Backbone	Atom-level	Fingerprint-level	Gain (%)
Binary	GCN	0.725	0.878	+21.1
	GIN	0.704	0.868	+23.3

Multilabel	GCN	0.623	0.759	+21.8
	GIN	0.631	0.753	+19.3

## Data Availability

The data used to support the findings of this study are available from the corresponding author upon request.
